# Modest radiation dose escalation for early-stage anal cancer: cancer control and toxicity outcomes

**DOI:** 10.3389/fonc.2025.1637205

**Published:** 2025-10-17

**Authors:** Sage Copling, Michael K. Rooney, Prajnan Das, Eugene J. Koay, Ethan B. Ludmir, Bruce D. Minsky, Sonal S. Noticewala, Grace L. Smith, Emma B. Holliday

**Affiliations:** ^1^ University of Texas Health Science Center Houston McGovern Medical School, Houston, TX, United States; ^2^ Department of Gastrointestinal Radiation Oncology, University of Texas MD Anderson Cancer Center, Houston, TX, United States

**Keywords:** anal cancer, local control, radiation dose response, toxicities, late effects

## Abstract

**Background:**

The optimal dose for patients with early-stage squamous cell carcinoma of the anus (SCCA) is unknown. We aimed to evaluate the impact of modest dose escalation (54 Gray (Gy)) compared with standard dose (50 Gray) on the disease-free survival and toxicity outcomes for patients with T1-2N0 SCCA.

**Methods:**

Patients with T1-T2N0 SCCA treated with definitive radiation from 1/1/2003 until 6/31/2022 were included in this retrospective analysis. Regression discontinuity analysis was performed to evaluate for a potential causal effect of modest dose escalation on freedom from local recurrence (FFLR). Cox proportional hazards model was generated to estimate the effect of modest dose escalation on FFLR, and an additional analysis was performed restricting the dataset to individuals with tumors measuring 1.5-2.5cm. Ordinal logistic regression was used to identify factors associated with several graded toxicity outcomes including acute and late gastrointestinal (GI), genitourinary (GU), dermatologic, and acute pain toxicities.

**Results:**

Two hundred thirty-four patients with T1N0 (N = 85, 36%) or T2N0 (N = 149, 64%) SCCA were included. Eighty-four (35.9%) received 50 Gy, 147 (62.8%) received 54Gy and 3 (1.3%) received 54–55 Gy. The median [IQR] time from the end of radiation to last follow up was 78 [44-119] months. Two- and 5-year FFLR were 90.7% and 88.6%. There was no significant association between modest dose escalation and FFLR (HR 0.6, 95% CI 0.2-1.9, P = 0.4) in patients with tumors 1.5-2.5 cm. In the global multivariable Cox regression model including all 234 patients, only positive HIV status (HR 5.2 [95% CI 1.2-21.5], P = 0.02) persisted as a significant predictor of worse FFLR on multivariate analysis. Modest dose escalation (P = 0.3) and tumor size (P = 0.6) did not predict FFLR. Modest dose escalation was associated with worse acute GU toxicity (OR 3.28, 95% CI 1.40-8.59, p = .01) and worse acute pain toxicity (OR 3.63, 95% CI 2.03- 6.65, p <.001).

**Conclusions:**

Modest dose escalation was not associated with improved FFLR among patients with T1-2N0 SCCA; however, it was associated with worse acute GU and pain toxicity. Future efforts should focus on biomarkers to identify patients who may potentially benefit from treatment escalation.

## 1 Introduction

For many decades, definitive chemoradiation (CRT) has been considered the standard of care for non-metastatic squamous cell carcinoma of the anus (SCCA). Cure rates are high, with 5-year disease-free survival rates (DFS) approaching 70% in randomized controlled studies UK ACT II and RTOG 9811 (1, 2). However, an analysis of local failure and overall survival for patients treated on RTOG 9811 suggests risk of locoregional failure is lower for patients with cT2N0 disease compared with more advanced stages. For this lowest risk group, 5-year locoregional failure was 17%, 3-year colostomy failure was 11%, 5-year DFS was 72% and 5-year overall survival (OS) was 82% (3).

The current standard of care radiation technique is intensity-modulated radiation therapy (IMRT) as established by a phase II trial RTOG 0529 (4). Patients with T1N0 disease were not eligible, and outcomes for patients with T2N0 disease were not reported separately. However, patients with T2N0 disease received a lower dose (50.4 Gray (Gy) in 28 fractions to the tumor with 42 Gy in 28 fractions to the elective nodal volume) compared with patients with T3–4 and/or node positive disease (54 Gy in 30 fractions to the tumor with 45 Gy in 30 fractions to the elective nodal volume) (4). Patients treated with IMRT-based CRT on RTOG 0529 also had a 5-year DFS of 70% but had significantly lower rates of grade 3+ acute dermatologic, grade 3+ acute gastrointestinal and grade 2+ acute hematologic toxicities compared with patients treated with conventional radiotherapy on RTOG 9811 (4, 5). Regarding late effects, 55% of patients treated on RTOG 0529 experienced late grade 2 toxicity and 16% experienced late grade 3 toxicity (5).

Finding the optimal balance between cure and treatment-induced toxicity has been the subject of much interest. The conformality and toxicity-sparing ability of IMRT allow for safer dose-escalation. An analysis of the National Cancer Database suggests there is considerable heterogeneity in dose selection for patients with T1-2N0 SCCA treated between 2004-2015. Out of 4,797 patients with T1-2N0 SCCA, 15% were treated to 45-<50 Gy, 27% were treated to 50–51 Gy and the remaining 58% were treated to >51–60 Gy. In that study, receipt of 54 Gy compared with 45-<54 Gy was associated with improved overall survival for patients with locally advanced SCCA (cT3–4 and/or N+), though there was no observed dose relationship among patients with early-stage disease (T1-2N0) (6).

Conversely, another line of investigation has been to determine whether safe radiation dose de-escalation may maintain excellent cure rates while improving toxicity and quality of life. PLATO ACT 4 randomized patients with T1-T2 (<4cm) N0 SCCA to either 41.4 Gy or 50.4 Gy, both delivered in 1.8 Gy fractions with concurrent capecitabine. Short-term results of this randomized phase 2 study showed excellent complete clinical response rates at six months for both the standard dose (87%) and reduced dose (92%) arms. Worse patient-reported sexual function was observed at six months after standard dose chemoradiation (7).

In the absence of clear data, it has been our institutional practice to prescribe 50 Gy in 25 fractions for T1N0 tumors and 54 Gy in 27 fractions for T2N0 tumors. In this analysis, we aimed to evaluate whether this modest dose escalation to 54Gy leads to improved disease control and/or if it leads to increased toxicity for patients with early stage SCCA. The following article was written accordance with the Strengthening the Reporting of Observational Studies in Epidemiology (STROBE) reporting checklist.

## 2 Methods

### 2.1 Patient population

The study was conducted in accordance with the Declaration of Helsinki (as revised in 2013). The study was approved by the MD Anderson Cancer Center institutional review board (protocol 2020-0513) and individual consent for this retrospective analysis was waived due to the retrospective nature of the study.

All consecutive patients at our institution with T1-T2N0 SCCA treated with definitive, IMRT-based radiation from 1/1/2003 until 6/31/2022 were included in this analysis.

### 2.2 Pretreatment evaluation

As is our institutional practice, all patients were seen by a multidisciplinary team including a colorectal surgeon, a medical oncologist, and a radiation oncologist. Pretreatment work up during the time period encompassed by this retrospective review included a digital rectal exam, endoscopic exam with anoscopy, proctoscopy or flexible sigmoidoscopy, and computed tomography (CT) scan of the chest, abdomen, and pelvis. A positron emission tomography (PET) scan and/or magnetic resonance imaging (MRI) pelvis were considered at the discretion of the treating physician and utilization of advanced imaging varied somewhat by time period. During the entire time period encompassed by the retrospective review, PET was not allowed by most insurance companies for patients with cT1N0 SCCA. Around 2016, our group shifted toward ordering baseline PET scans on patients with T2N0 tumors and occasionally T1N0 when allowed by insurance. Our group did not routinely order pelvic MRI scans for patients with T1-T2N0 tumors until 2022. Some patients arrived at their initial appointment at our institution with PET or MRI scans ordered by their previous care team. If available, the advanced imaging studies were utilized in the treatment planning process, however measurement of the primary tumor for the purposes of clinical staging was performed by the colorectal surgeon on initial clinical and endoscopic exam.

### 2.3 Radiation treatment details

All patients underwent a CT simulation for treatment planning and were treated with definitive chemoradiation using an IMRT technique. A simultaneous integrated boost was used with two target volumes. The anal primary tumor target gross tumor volume (GTVp) was delineated based on physical exam, proctoscopy and/or CT/PET scans. The clinical target volume (CTVp) included a 1cm expansion, and the dose and fractionation were selected based on size. Our institutional practice was to treat T1 tumors to 50Gy in 25 fractions and T2 tumors to 54Gy in 27 fractions. The elective nodal CTV (CTVen) included the internal iliac, external iliac, perirectal, presacral, obturator and inguinal lymph nodes. The elective dose was 43Gy in 25 fractions when treating T1 tumors and 45Gy in 27 fractions when treating T2 tumors. The planning target volume (PTV) for all targets included a 5mm expansion to account for set up uncertainty. Most patients treated prior to 2015 were treated with a static field IMRT, while most patients treated in 2015 or later were treated with dynamic volumetric modulated arc therapy (VMAT).

### 2.4 Chemotherapy treatment details

Concurrent chemotherapy was given at the discretion of the treating medical oncologist. The majority of patients received weekly cisplatin (20mg/m^2^ on day 1 of each week) and daily 5FU (300mg/m^2^ infused continuously Monday through Friday of each week). An alternative regimen included mitomycin C (10mg/m^2^ on day 1 and day 28) instead of cisplatin. A minority of patients received capecitabine (825mg/m^2^ twice daily Monday through Friday) instead of 5FU. A small number of patients received radiation alone for resected T1N0 disease and/or the inability to tolerate chemotherapy.

### 2.5 Toxicity assessment and follow-up

During treatment, patients were seen weekly by their radiation oncologist. Acute radiation-related toxicities (gastrointestinal, genitourinary, dermatologic and pain) were assessed using the Common Terminology Criteria for Adverse Events version 4 [CTCAEv4 (8)] and documented weekly in the medical record by their radiation oncologist. After completion of chemoradiation, patients returned for follow up every three months until year two, every six months until year four, and then annually. Digital rectal exam was performed at each follow up. Endoscopic exam was performed every three months until complete clinical response was documented and then every six months for two years post-treatment. Cross sectional imaging was performed every three months until complete clinical response was documented and then annually for five years. Cross sectional imaging always included a CT chest, abdomen and pelvis but could also include a PET and/or MRI pelvis at the discretion of the treating physician. Toxicities documented up to six weeks post-completion of chemoradiation were recorded as acute toxicities. Toxicities reported after that time point were recorded as late toxicities.

### 2.6 Statistical analysis

Descriptive statistics were used to summarize the population characteristics. Median values with value ranges were used to describe continuous variables. Non-parametric testing was used to compare differences across groups. Modest dose escalation was defined as >/=54 Gy.

The primary oncologic endpoint of interest was freedom from local recurrence (FFLR), defined as the time in months from completion of CRT to local recurrence. For patients who did not attain a complete clinical response after CRT, date of recurrence was defined as the date of clinical progression or the date of clinically confirmed persistent disease at least six months after treatment completion. Patients who were alive without evidence of disease at last follow up were censored and non-informative censoring was assumed.

Multiple modelling strategies were utilized to estimate the impact of various predictive covariates on FFLR. First, a regression discontinuity analysis was performed to evaluate for a potential causal effect of dose escalation on FFLR. The running predictive variable was tumor size, which per institutional standard practice, is used as a primary factor to determine radiation dose strategies. Patients with tumors less than 2cm (T1 disease) are typically treated with 50 Gy, while those with tumors from 2-5cm (T2 disease) are typically treated with dose escalated radiation consisting of 54 Gy. Thus, a tumor size of 2 cm was used as the cutoff for predicting radiation dose escalation. The assumption of a sharp regression discontinuity (i.e., perfect treatment allocation based on the running variable) was evaluated graphically. A local Cox proportional hazards model was generated, including only patients with tumors within the selected cutoff window, to estimate the effect of modest dose escalation on FFLR. Given the fuzzy nature of radiation dose selection, an interaction instrument was used to improve treatment effect estimation.

To further assess the potential treatment effect of radiation dose on FFLR, a multivariable Cox regression model was constructed using all patients with T1-T2N0 disease in the cohort. Potential predictive covariates included sex, race, ethnicity, HIV status, tumor size, tumor resection status, age, and modest radiation dose escalation. Other covariates with small subgroups or limited numbers of events were excluded from the final model. Factor selection was performed using univariable Cox regression, retaining covariates with P<0.1 in the final model. Survival outcomes were visualized with the Kaplan-Meier method.

Ordinal logistic regression was used to identify factors associated with several graded toxicity outcomes including acute and late gastrointestinal (GI), genitourinary (GU), dermatologic, and acute pain toxicities. All toxicities were measured on a scale of 0 to 5, with increasing scores reflecting worse toxicity. Potential predictive covariates included sex (categorical), race (categorical), ethnicity (categorical), HIV status (categorical), tumor size (cm) (continuous), excision prior to RT (binary), chemotherapy regimen used (categorical), age at radiation start (continuous), and modest radiation dose escalation (50 Gy vs 54 Gy). Factor selection was performed using both forward and backward stepwise selection with minimization of the Akaike Information Criterion (AIC) to determine optimal models. Radiation dose escalation was manually included in final models if it was not selected for previously, as this was a covariate of interest.

All statistical analyses were conducted using R 4.1.1 (R Foundation for Statistical Computing, Vienna, Austria). Statistical significance was defined as P < 0.05 (two-sided).

## 3 Results

### 3.1 Patient population

Two hundred thirty-four patients with T1N0 (N = 84, 35.9%) or T2N0 (N = 150, 64.1%) SCCA were included in the analysis. While all patients were staged with initial CT chest, abdomen and pelvis, a digital rectal exam and an endoscopic exam by a colorectal surgeon, 45 (19.2%) underwent initial staging MRI pelvis and 107 (45.7%) underwent initial staging PET. Patient, tumor, and treatment characteristics are outlined in **Table 1**. There were some notable differences between the patients treated with modest dose escalation and those treated with standard dose; patients treated with modest dose escalation more often had T2 tumors and a larger tumor size but less often had at least a partial excision prior to radiation. The median [IQR] duration of treatment was 36.5 [34.5-37.5] days, and 23 (9.8%) required a treatment break. The median [IQR] time from the end of radiation to last follow up was 78 [44-119] months. All patients were followed with digital rectal exams and endoscopic exams. With regard to cross-sectional imaging, 193 (82.5%) were followed with yearly CT CAP alone once they attained complete clinical response and 41 (17.5%) received surveillance PET and/or pelvic MRI during the follow up period.

**Table 1:** Patient, tumor, and treatment characteristics for patients with T1-2N0 squamous cell carcinoma of the anus included in this cohort.

Characteristic	Total cohort (N = 234)	50-50.4Gy (N = 84, 35.9%)	54-55Gy (N = 150, 64.1%)	P-value
Sex				
Female	183 (78.2%)	66 (78.6%)	117 (78%)	.919
Male	51 (21.8%)	18 (21.4%)	33 (22%)	
Race				
White	221 (94.4%)	82 (97.6%)	139 (92.7%)	.113
Non-White	13 (5.6%)	2 (2.4%)	11 (7.3%)	
Ethnicity				
Non-Hispanic or Latino	223 (95.3%)	82 (97.6%)	141 (94%)	.210
Hispanic or Latino	11 (4.7%)	2 (2.4%)	9 (6%)	
Age at RT (Median, IQR)	62.5 (56.0-69.0)	62.2 (56.1-69.0)	62.7 (56.4-68.5)	.749
HIV status				
Negative	228 (97.4%)	83 (98.8%)	145 (96.7%)	.320
Positive	6 (2.6%)	1 (1.2%)	5 (3.3%)	
T-stage				
T1	85 (36.3%)	73 (86.9%)	12 (8%)	<.001
T2	149 (63.7%)	11 (13.1%)	138 (92%)	
Size of Anal Tumor in cm (Median, IQR)	2.5 (1.7- 3.0)	1.5 (1.2-2)	3 (2.6-3.5)	<.001
Excision prior to RT				
None, biopsy only	162 (69.2%)	41 (48.8%)	121 (80.7%)	<.001
At least partial excision	72 (30.8%)	43 (51.2%)	29 (19.3%)	
Chemotherapy agents				
None	6 (2.6%)	1 (1.2%)	5 (3.3%)	.677
Cisplatin/5FU	183 (78.2%)	65 (77.4%)	118 (78.7%)	
MMC/5FU	36 (15.4%)	15 (17.9%)	21 (14%)	
5FU/Capecitabine Monotherapy	9 (3.8%)	3 (3.6%)	6 (4%)	

### 3.2 Oncologic outcomes

In the entire cohort of 234 patients, the 2- and 5-year FFLR were 90.7% (95% CI 87.0%-94.6%) and 88.6% (95% CI 84.4%-92.9%), respectively. The 2- and 5-year colostomy-free survival rates were 93.3% (95% CI 90.1%-96.6%) and 90.1% (95% CI 86.1%-94.2%), respectively. The 2- and 5-year distant failure-free survival were 96.5% (95% CI 94.2%-98.9%) and 92.7% (95% CI 89.2%-96.4%), respectively. The 2- and 5-year overall survival were 95.2% (95% CI 92.5%-98.0%) and 88.7% (95% CI 84.6%-93.1%), respectively.

### 3.3 Regression discontinuity analysis

**Figure 1:**
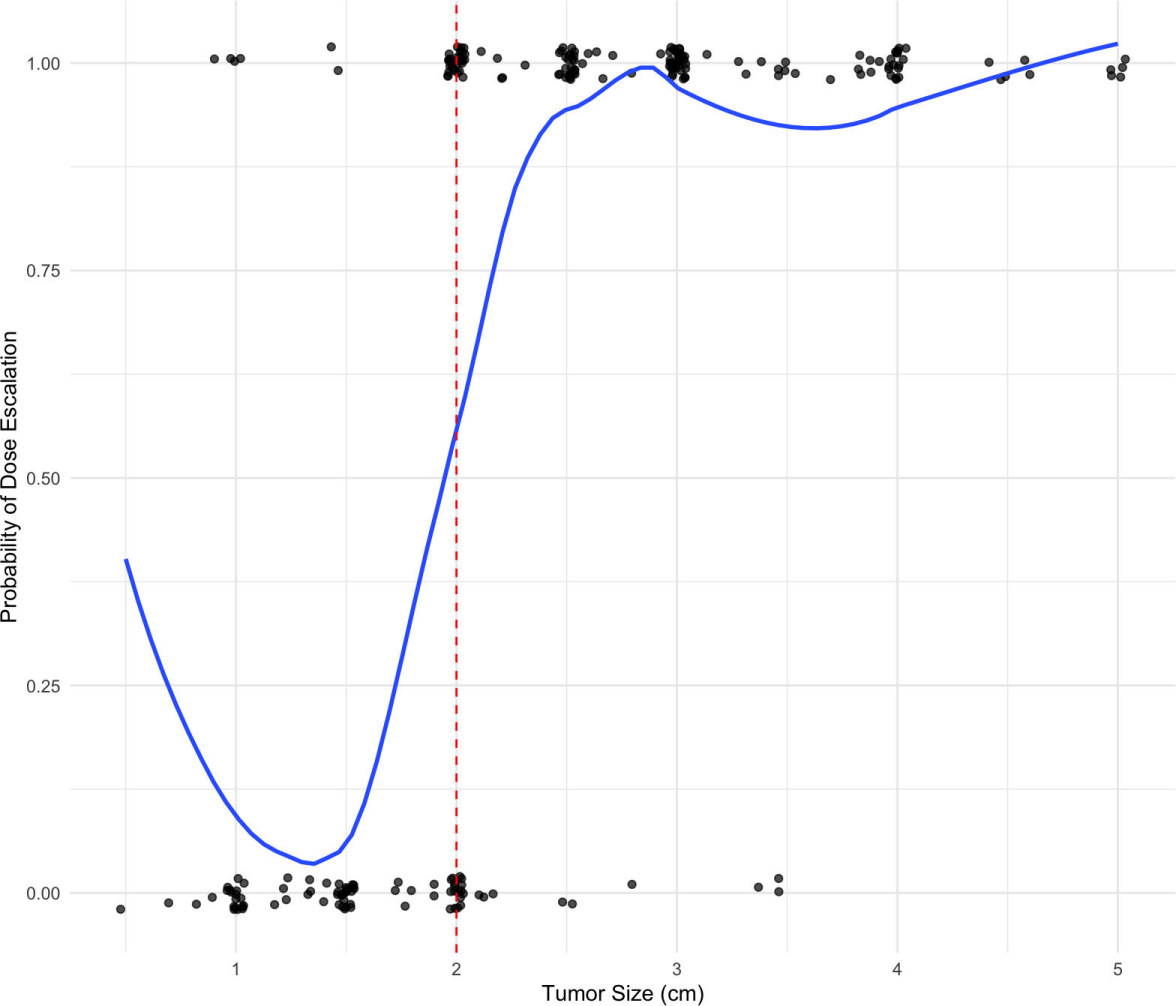
Relationship between tumor size and radiation dose escalation.

The assumption of treatment allocation based on a running continuous variable with defined cut point was evaluated graphically in **Figure 1**. Here, the treatment of interest is modest radiation dose escalation. As displayed in the figure, dose escalation was strongly predicted by tumor size with the expected cutoff at 2cm. However, radiation dose escalation was not perfectly predicted by tumor size and thus a fuzzy regression discontinuity design was adopted.

Several local window sizes around the 2 cm cutoff were analyzed, and a 5 mm window was selected to balance predictive ability with sample size. To assess the validity of the regression discontinuity design as a quasi-experimental randomization tool, characteristics of patients with tumor sizes locally above and below the cutoff were compared (1.5–2 cm vs. 2 cm vs. 2–2.5 cm). As summarized in **Table 2**, The groups were comparable, supporting the appropriateness of the fuzzy regression discontinuity design.

**Table 2:** Characteristics of patients with T1-2N0 tumors within the selected 0.5 window around the 2cm treatment allocation cutoff.

Characteristic	Primary tumor size		
	2.1 – 2.5cm	2.0cm	1.5cm – 1.9cm
	(N = 36)	(N = 44)	(N = 29)
Sex			
Female	30 (83.3%)	38 (86.4%)	22 (75.9%)
Male	6 (16.7%)	6 (13.6%)	7 (24.1%)
Race			
Black	1 (2.8%)	1 (2.3%)	1 (3.4%)
White	35 (97.2%)	43 (97.7%)	28 (96.6%)
Ethnicity			
Hispanic or Latino	1 (2.8%)	6 (13.6%)	0 (0%)
Non-Hispanic/Latino	35 (97.2%)	38 (86.4%)	29 (100%)
Age (years)			
Mean (SD)	63.3 (9.95)	60.3 (11.6)	63.0 (8.30)
Median [Min, Max]	63.0 [49.0, 89.0]	61.0 [33.0, 78.0]	63.0 [48.0, 82.0]
HIV status			
No	35 (97.2%)	44 (100%)	29 (100%)
Yes	1 (2.8%)	0 (0%)	0 (0%)
Tumor grade			
1	3 (8.3%)	7 (15.9%)	3 (10.3%)
2	16 (44.4%)	16 (36.4%)	15 (51.7%)
3	15 (41.7%)	19 (43.2%)	10 (34.5%)
Unknown	2 (5.6%)	2 (4.5%)	1 (3.4%)
Resection prior to radiation			
None, biopsy only	27 (75.0%)	30 (68.2%)	17 (58.6%)
At least partial excision	9 (25.0%)	14 (31.8%)	12 (41.4%)
Chemotherapy agents			
None	1 (2.8%)	1 (2.3%)	1 (3.4%)
Cis-5FU	24 (66.7%)	35 (79.5%)	24 (82.8%)
MMC-Cape	7 (19.4%)	5 (11.4%)	4 (13.8%)
5FU-Cape	4 (11.1%)	3 (6.8%)	0 (0%)

**Figure 2:**
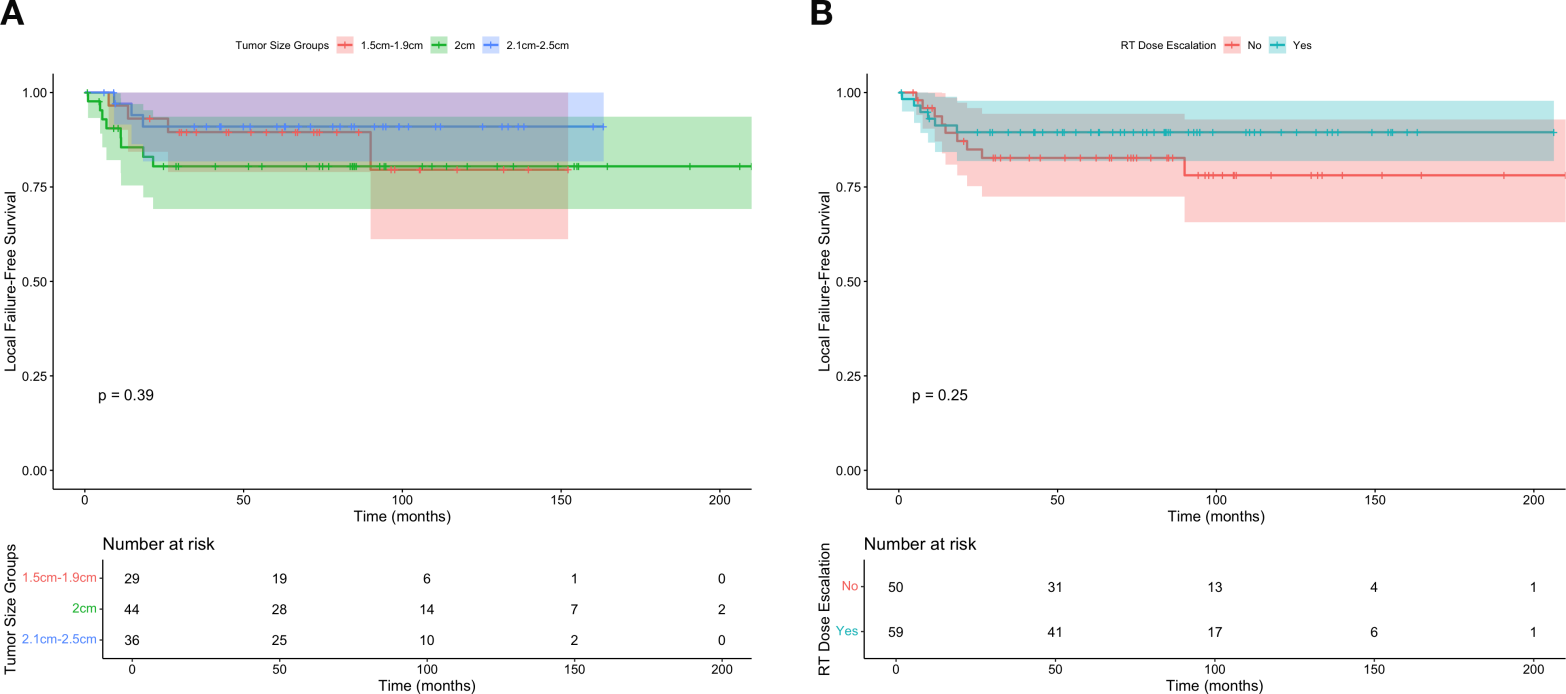
Freedom from local failure for individuals with T1-2N0 squamous cell carcinoma of the anus within the regression discontinuity cutoff widow according to **(A)** tumor size and **(B)** receipt of dose escalated radiation (defined as 54 Gray).

A local Cox proportional hazards model was generated to estimate the effect of dose escalation on FFLR, restricting the dataset to individuals with tumors within the selected 0.5cm cutoff window (e.g., tumors from 1.5-2.5cm). In this model, there was no significant association between dose escalation and FFLR (HR 0.6, 95% CI 0.2-1.9, P = 0.4). These results are further visualized in **Figure 2**, which similarly shows no significant differences in FFLR among the restricted population, with stratification either by tumor size or radiation dose escalation.

### 3.4 Cox proportional hazards modelling for FFLR

Using all patients with T1-2N0 disease in the cohort, we additionally created a multivariable Cox proportional hazards model to estimate factors associated with FFLR. Univariate comparisons were performed first, and factors associated with FFLR (P<0.1) were included in the final model. A summary of all univariate comparisons is provided in **Table 3**; the only significant predictors were sex and HIV status. As dose escalation and tumor size were variables of interest for this investigation, we included those as additional factors in the final multivariable model, summarized in **Table 3**. In the final model, only positive HIV status (HR 5.2 [95% CI 1.2-21.5], P = 0.02) persisted as a significant predictor of worse FFLR. Sex (P = 0.2), dose escalation (P = 0.3) and tumor size (P = 0.6) did not predict FFLR.

**Table 3:** Univariable and multivariable Cox proportional hazards model predicting FFLR, including all patients with T1-2N0 disease in this cohort.

Characteristic	Univariable model			Multivariable model		
	Hazard ratio	95% Confidence interval	P value	Hazard ratio	95% Confidence interval	P value
Sex						
Female	Ref			Ref		
Male	2.2	1.0-4.7	0.05	1.7	0.7-4.2	0.2
Race						
Non-White	Ref					
White	1.5	0.2-11	0.7			
Ethnicity						
Hispanic or Latino	Ref					
Non-Hispanic/Latino	0.6	0.1-2.4	0.4			
Age (years, continuous)	0.99	0.98-1.0	0.4			
HIV status						
No	Ref			Ref		
Yes	5.5	1.6-18.3	0.005	5.2	1.2-21.5	0.02
Tumor Size (cm)	0.8	0.5-1.2	0.3	0.9	0.5-1.5	0.6
Resection status						
Biopsy Only	Ref					
R0	0.3	0.04-2.5	0.3			
R1	0.5	0.2-1.8	0.3			
R2	NA	NA	NA			
Dose escalation						
No	Ref			Ref		
Yes	0.6	0.3-1.2	0.2	0.6	0.2-1.6	0.3

NA (Not Applicable) signifies that outcome could not be reported by the model due to low incidence in the population.

### 3.5 Acute toxicities

For acute gastrointestinal (GI) toxicity, 73 (31.2%) developed G2 toxicity, 20 (8.5%) developed G3 toxicity, five (2.1%) developed G4 toxicity and 1 (0.4%) developed G5 toxicity. For acute genitourinary (GU) toxicity, 28 (12.0%) developed G2 toxicity and 5 (2.1%) developed G3 toxicity. For dermatologic toxicity, 155 (66.2%) developed G2 toxicity and 36 (15.4%) developed G3 toxicity. For acute pain toxicity, 118 (50.4%) developed G2 toxicity and 19 (8.1%) developed G3 toxicity.

After forward and backward factor selection of ordinal logistic regression variables for acute GI toxicities, only dose escalation was identified as the primary predictor of toxicity, though this relationship was not significant (OR of 0.67, 95% CI 0.41-1.10, P = 0.11) (**Table 4**).

**Table 4:** Ordinal logistic regression model predicting acute and late physician-graded toxicity according to the CTCAE v4, including all patients with T1-2N0 disease in this cohort.

Toxicity type	Predictor	OR	95% CI	P-value
Acute Pain Toxicity	Dose Escalation (Binary, 54 Gy vs. ≤50.4 Gy)	3.63	2.03 - 6.65	< 0.001
	Age at RT Initiation (years)	0.96	0.93 - 0.98	0.002
	Excision Prior to RT (vs. No excision)	1.79	0.94 - 3.44	0.08
	Hispanic/Latino Ethnicity (vs. non- Hispanic/Latino)	0.41	0.12 - 1.37	0.15
Acute GI Toxicity	Dose Escalation (Binary, 54 Gy vs. ≤50.4 Gy)	0.67	0.41 - 1.10	0.11
Late GI Toxicity	Dose Escalation (Binary, 54 Gy vs. ≤50.4 Gy)	1.22	0.71 - 2.13	0.47
	Cisplatin/5-FU Chemotherapy (vs. None)	1.65	0.34 - 11.94	0.56
	Mitomycin/5-FU Chemotherapy (vs. None)	0.74	0.13 - 5.87	0.74
	5-FU/Capecitabine Monotherapy (vs. None)	0.32	0.01 - 4.35	0.40
	Hispanic/Latino Ethnicity (vs. non- Hispanic/Latino)	0.28	0.04 - 1.12	0.11
Acute GU Toxicity	Dose Escalation (Binary, 54 Gy vs. ≤50.4 Gy)	3.28	1.40 - 8.59	0.01
	Male Sex (vs. Female)	3.16	1.47 - 6.71	< 0.001
	Excision Prior to RT (vs. No excision)	2.20	0.94 - 5.03	0.06
Late GU Toxicity	Dose Escalation (Binary, 54 Gy vs. ≤50.4 Gy)	2.18	0.83 - 6.80	0.14
Acute Dermatologic Toxicity	Dose Escalation (Binary, 54 Gy vs. ≤50.4 Gy)	1.12	0.53 - 2.36	0.76
	Excision Prior to RT (vs. No excision)	2.27	1.18 - 4.41	0.01
	Hispanic/Latino Ethnicity (vs. non- Hispanic/Latino)	0.38	0.11 - 1.32	0.12
	Tumor Size (cm)	1.33	0.93 - 1.89	0.12
Late Dermatologic Toxicity	Dose Escalation (Binary, 54 Gy vs. ≤50.4 Gy)	N/A	N/A	N/A

*Dermatologic late toxicity only occurred 3 times in this dataset, so ordinal logistic regression could not be reported.

For acute GU toxicities, the following variables were reported as the primary predictors of toxicity: dose escalation (OR 3.28, 95% CI 1.40-8.59, P = 0.01), male sex (OR 3.16, 95% CI 1.47- 6.71, P = < 0.001), and excision of the tumor prior to radiation (OR: 2.20, 95% CI 0.94-5.03, P = 0.06) (**Table 4**).

For acute dermatologic toxicities, excision prior to RT was the only variable with a significant relationship to acute dermatologic toxicities (OR: 2.27, 95% CI 1.18-4.41, P = 0.01) (**Table 4**).

For acute pain toxicity, we observed a significant association with radiation dose escalation (OR 3.63, 95% CI 2.03- 6.65, p < 0.001). Age had a significant negative association with acute pain toxicity (OR: 0.96, 95% CI 0.93-0.98, P = 0.002) (**Table 4**).

### 3.6 Late toxicities

For late GI toxicity, 34 (14.5%) developed G2 toxicity, six (2.6%) developed G3 toxicity and three (1.3%) developed G4 toxicity. For late GU toxicity, 11 (4.7%) developed G2 toxicity, one (0.4%) developed G3 toxicity and two (0.9%) developed G4 toxicity. For late dermatologic toxicity, only one (0.4%) patient developed G2 toxicity.

Dose escalation was not found to have a significant relationship with late GI, GU, or dermatologic toxicities in this dataset and nor were any of the clinical or treatment variables assessed (**Table 4**).

## 4 Discussion

In this retrospective analysis of 234 patients with T1-2N0 SCCA treated at a single institution, we found no significant association between dose escalation to 54Gy and improved FFLR. However, we did observe increased acute pain toxicity and increased acute GU toxicity among patients treated with dose escalation to 54 Gy.

Consistent with prior published work, patients in our cohort treated for T1-2N0 SCCA did well with regard to FFLR, colostomy-free survival, distant failure-free survival and overall survival. Patients with T2N0 disease treated on RTOG 9811 had 83% freedom from locoregional failure, 89% freedom from colostomy failure, 90% freedom from distant metastases and 82% overall survival at five years (3). Patients with T1N0 disease treated with radiation in the National Cancer Database have reported 5-year OS of 86.8%, though other oncologic outcomes are not available in this database (9). Our five-year outcomes compare favorably at 88.6% 5-year FFLR, 90.1% CFS, 92.7% DFFS and 88.7% OS.

Our study is unique in that it analyzed outcomes between patients receiving 50 Gy and modest dose escalation of 54 Gy. Most studies evaluating dose escalation utilized total tumor doses around or in excess of 60 Gy. RTOG 9208 evaluated a split-course regimen to 59.4 Gy with concurrent 5FU and mitomycin C in a cohort of 47 patients with SCCA >/=2cm. They did not find improved local control compared with patients treated on RTOG 8704 with 45-50.4 Gy followed by a 9 Gy only for patients with residual disease on post-treatment biopsy (10). A retrospective analysis by Ferrigno et al. showed patients treated to a dose >50 Gy had improved local control, but with an overall sample size of 43 patients, few had T1-2N0 disease (11). The CORS 03 retrospective study also utilized a split-course approach, in which patients received a mean of 45 Gy followed by boost with either external beam (mean 18 Gy) or brachytherapy techniques. In this study, the majority of patients had T1 (19%) or T2 (48%) tumors. They found 5-year cumulative rate of local recurrence was improved among patients who received brachytherapy boost, as long as the overall treatment time was kept to <80 days (12).

The strategy of dose escalation has been explored mostly for patients with more locoregionally advanced disease. However, some studies have included patients with earlier stage disease. The ACCORD 3 study included a small proportion of patients with T2N0 SCCA with tumors >/=4cm and randomized them to a total of 60 Gy or 70–75 Gy to the primary tumor with concurrent 5FU and cisplatin. The primary endpoint of this study was CFS, and they did not show improved CFS with higher-dose boost. They showed a small and nonsignificant improvement for LC at 3 and 5 years with 70–75 Gy compared with 60 Gy (84%, 83.1% vs. 79%, 78.2%, respectively; P = 0.28), but overall control rates were high for this higher risk cohort of patients (13). Another modern study from University Hospital Muenster utilized 3D conformal RT or IMRT to give escalated dose (median 63 Gy) to 87 patients with SCCA; 24% of patients had T1 disease, 36% of patients had T2 disease and 50% of patients had N0 disease. The 3-year CFS was quite high in this study at 97%, and they found a significant improvement in colostomy-free survival for patients with T2/T3 tumors with dose escalation > 63 Gy. The 3-year PFS in this study was high at 78.5%, and they found improved progression-free survival with >63 Gy for patients with T1/T2 tumors, but the number of patients in this subset was small (N = 41) (14).

Our results show modest dose escalation of 54 Gy to the anal canal is associated with increased acute toxicity compared with 50 Gy, particularly acute genitourinary toxicity, and acute pain during and for up to six weeks after treatment. This makes sense given the radiosensitive mucosa of the anal canal and painful nature of perianal dermatitis. The GU toxicities observed in our study were primarily irritative bladder symptoms such as frequency or urgency of urination or obstructive lower urinary tract symptoms (LUTS) such as difficulty urinating and weak stream. While irritative bladder symptoms were seen among men and women, obstructive LUTS were seen mostly among men due to the impact of prostate inflammation during pelvic radiation (15). Interestingly, we did not see increased rates of late toxicities in our study for patients treated with 54 Gy compared with those treated to 50 Gy, but this may be due to inconsistent recording of late toxicities in our database as opposed to acute toxicities which are recorded by the treating physician in a templated manner. The University Hospital Muenster series did not show a significant difference in overall acute toxicities between those treated >63 Gy and those treated <63 Gy, but they did show a higher rate of chronic skin toxicities (43.8% vs. 69%, P = 0.042) (13).

United States and European guidelines cite ranges of acceptable doses supported by literature rather than one specific dose and fractionation regimen (16, 17). National database studies likewise suggest considerable variation in dose selection and suggest no significant association between increased radiation dose and increased survival (6). Recently published guidelines from the American Society of Radiation Oncology strongly recommended doses of 45-50.4 Gy for T1-2N0 tumors, although they note higher doses may be reasonable for tumors >/=4cm (18).

Some argue 45-50.4 Gy may even be too high a dose for early-stage anal cancer. Indeed, ongoing and recently completed randomized trials explore dose de-escalation as a strategy to optimize the therapeutic ratio. PLATO ACT 3 is a non-randomized trial evaluating de-escalated treatment for T1N0 SCCA treated with local excision in which patients with negative margins are observed while patients with margins </=1mm are treated with 41.4 Gy with concurrent capecitabine. Moreover, ECOG-ACRIN trial 2182 (DECREASE) includes patients with T1-T2(<4cm) N0 disease and randomizes them to 50.4 Gy in the standard of care arm and 41.4 Gy (T2) or 36 Gy (T1) in the dose de-escalated arm (19). Results from these studies are eagerly anticipated.

Short-term results from PLATO ACT 4 were recently published (7). They found no substantial difference in clinical complete response rates between those randomized to 41.4 Gy vs 50.4 Gy (both rates >85%), while reporting a higher rate of serious (Grade 3 or greater) adverse events and worse long-term patient-reported sexual function among the patients who received standard (higher) dosing Moreover, patient reported outcomes were reported regarding sexual function arising in the group receiving higher doses of radiation. Finally, both chemotherapy and radiation therapy interruption rates were both higher in the group receiving the higher radiation dose, potentially indicating that toxicities associated with higher doses in this patient population may impact overall treatment tolerance and adherence (7). Between ACT 4 and the cohort described in this retrospective analysis, we find baseline characteristic to be similar in terms of average age, HIV status, and gender. While the dose difference reported in the ACT 4 trial is more drastic than we report in this analyses, we report similar findings of limited survival and local control benefits from elevation of radiation dose in patients with T1-T2/N0 SCCA, in addition to a worsened toxicity profile. Our cohort is slightly bigger, however the retrospective nature of this analysis limits its generalizability.

As mentioned, one major limitation in our dataset is the lack of complete and standardized late toxicity reporting, which could potentially contribute to underestimation of late toxicity. Not all patients received the same diagnostic workup, which is a limitation in the interpretability of our findings with regards to staging and sizing. Other limitations include the retrospective nature of this study and the potentially unmeasured and situation-specific factors that may lead the treating physician to have chosen 50 Gy or 54 Gy for any given patient. Indeed, patients for whom modest dose escalation was selected more often had T2 tumors and larger tumor size and less often had at least a partial excision before definitive radiation. Although our use of a statistical model restricting the dataset to individuals with tumors within the selected 0.5cm cutoff window (e.g., tumors from 1.5-2.5cm) helped to partially mitigate this bias, it cannot obviate it completely. Another limitation of this study is that there were no patients with >4 cm tumors who received 50 Gy, so results should be applied with caution to patients with larger T2N0 tumors, potentially limiting the generalizability of our results in this population of patients. Finally, this study is limited by the lack of patient-reported outcome measures of toxicity and function.

## 5 Conclusions

In conclusion, the results from our study have led us to change our institutional practice and utilize 50 Gy as our standard prescription dose for patients with T1-2N0 SCCA. Modest dose escalation to 54 Gy does not appear to benefit most patients, but future work is needed to identify reliable biomarkers for higher risk of recurrence. Human papillomavirus circulating tumor DNA is a promising biomarker that may identify patients who could benefit from treatment escalation, whether from radiation dose escalation or systemic therapy escalation, in the near future (20).

## Data Availability

The raw data supporting the conclusions of this article will be made available by the authors, without undue reservation.
